# Effects of varenicline and cognitive bias modification on neural response to smoking-related cues: study protocol for a randomized controlled study

**DOI:** 10.1186/1745-6215-15-391

**Published:** 2014-10-07

**Authors:** Angela S Attwood, Tim Williams, Sally Adams, Francis J McClernon, Marcus R Munafò

**Affiliations:** Medical Research Council Integrative Epidemiology Unit, University of Bristol, 12a Priory Road, Bristol, BS8 1TU UK; UK Centre for Tobacco and Alcohol Studies, University of Bristol, 12a Priory Road, Bristol, BS8 1TU UK; School of Experimental Psychology, University of Bristol, 12a Priory Road, Bristol, BS8 1TU UK; Department of Psychology, 2 South, University of Bath, Bath, BA2 7AY UK; Psychiatry and Behavioral Sciences, Duke University School of Medicine, Hock Building, Box 2701, 2424 Erwin Road, Durham, NC 27705 USA

## Abstract

**Background:**

Smoking-related cues can trigger drug-seeking behaviors, and computer-based interventions that reduce cognitive biases towards such cues may be efficacious and cost-effective cessation aids. In order to optimize such interventions, there needs to be better understanding of the mechanisms underlying the effects of cognitive bias modification (CBM). Here we present a protocol for an investigation of the neural effects of CBM and varenicline in non-quitting daily smokers.

**Methods/Design:**

We will recruit 72 daily smokers who report smoking at least 10 manufactured cigarettes or 15 roll-ups per day and who smoke within one hour of waking. Participants will attend two sessions approximately one week apart. At the first session participants will be screened for eligibility and randomized to receive either varenicline or a placebo over a seven-day period. On the final drug-taking day (day seven) participants will attend a second session and be further randomized to one of three CBM conditions (training towards smoking cues, training away from smoking cues, or control training). Participants will then undergo a functional magnetic resonance imaging scan during which they will view smoking-related pictorial cues. Primary outcome measures are changes in cognitive bias as measured by the visual dot-probe task, and neural responses to smoking-related cues. Secondary outcome measures will be cognitive bias as measured by a transfer task (modified Stroop test of smoking-related cognitive bias) and subjective mood and cigarette craving.

**Discussion:**

This study will add to the relatively small literature examining the effects of CBM in addictions. It will address novel questions regarding the neural effects of CBM. It will also investigate whether varenicline treatment alters neural response to smoking-related cues. These findings will inform future research that can develop behavioral treatments that target relapse prevention.

**Trial registration:**

Registered with Current Controlled Trials: ISRCTN65690030. Registered on 30 January 2014.

## Background

Smoking remains a significant public health concern, with tobacco-related deaths being estimated at around 6 million per year [1]. Despite many smokers reporting wanting to quit, few achieve long-term abstinence. Many episodes of drug relapse, including relapse to smoking, occur in the presence of drug-related cues [2, 3]. Through repeated and contingent pairing with drug administration, these cues acquire powerful motivational properties that can precipitate craving and drug-seeking [4–7]. Drug-related cognitive biases, characterized by selective or disproportionate attention allocation to drug cues, have been reported in users of a number of drugs and have been positively associated with drug craving [8, 9], future drug use [10], approach behaviors to drug-related cues [11], and increased likelihood of relapse [12]. Of particular importance, the drug-stimulus learning that is believed to underlie these biases is long-lasting, which makes an individual vulnerable to relapse long after initial cessation. In smokers, increased reactivity to smoking cues has been found to predict decreased likelihood of cessation [13, 14]. Consequently, reduction in cognitive bias is considered a promising target for therapeutic intervention.

Recent research indicates that it is possible to reduce cognitive biases using computer-based cognitive bias modification (CBM) paradigms that train individuals to allocate attention away from disorder-relevant cues. CBM has been shown to reduce cognitive biases in anxiety and depression [15–17] and has also been associated with reduction in other symptoms [18]. Attwood *et al*. [19] investigated the effect of a single session of stimulus-avoidant CBM using a modified dot-probe task, and reported decreases in cognitive bias in daily smokers. Compared to a group who had been trained to attend to smoking cues, there was evidence that the avoid group also showed attenuated craving in response to *in vivo* smoking cues in a subsequent cue exposure test (male participants only). The current study will extend earlier work by investigating the neural responses to smoking-related cues following CBM. The neuroimaging literature suggests that drug-related cognitive biases are the results of a failure of cognitive regulatory systems to increase control in the presence of salient cues that increase processing in the reward and emotional centers of the brain (such as the striatum and amygdala) [20]. These data will enable the investigation of whether CBM decreases emotional responses to drug-related cues or increases cognitive control in their presence, and will thereby increase understanding of the mechanisms that underlie CBM effects.

There has been a recent suggestion that the smoking cessation pharmacotherapy varenicline reduces cue-induced craving and cue-induced reinstatement of drug-taking in animals [21–24]. This study will include a pharmacological challenge that randomizes participants to receive seven days treatment of either varenicline or placebo prior to a functional Magnetics Resonance Imaging (fMRI) scan, in order to assess neural cue-reactivity following varenicline treatment. Previous research has reported differences in neural response to smoking cues following varenicline treatment [25], but the sample sizes were small and the effects require replication.

### Study objectives and hypotheses

The primary objective is to investigate the neural effects of CBM in response to smoking-related cues in current daily smokers. A secondary objective is to examine the effects of varenicline treatment on neural responses to smoking cues. We will also explore whether there is any pattern in the data to suggest that the effects of CBM are modified by concurrent treatment with varenicline. The CBM methods we will use are delivered via computer, and can therefore in principle be delivered remotely and repeatedly at low cost, for example via the internet or smartphones, as an adjunctive treatment to varenicline pharmacotherapy. To investigate whether CBM and varenicline effects generalize to other measures of cognitive bias, participants will also complete a modified pictorial Stroop task before and after CBM and varenicline.

We hypothesize that: 1) CBM designed to induce cognitive bias towards smoking-relates cues will lead to an increase in cognitive bias and neural response to smoking-related cues in brain regions previously implicated in cue reactivity in cigarette smokers; 2) CBM designed to induce cognitive bias away from smoking-relates cues will lead to a decrease in cognitive bias and neural response to smoking-related cues in brain regions previously implicated in cue reactivity in cigarette smokers; and 3) varenicline treatment will lead to a reduction in the neural response to smoking-related cues.

## Methods/Design

### Trial design

This is a human laboratory study assessing behavioral and neural outcomes of CBM. The study design is a two × three between-subjects model with one factor of drug (varenicline or placebo) and one factor of CBM group (attend, avoid, or control). For the behavioral assessments of cognitive bias (dot-probe and modified Stroop) and cue reactivity (cue exposure task in scanner), there will be an additional within-subjects factor of cue type (smoking or neutral).

### Participants and recruitment

**Figure 1:**
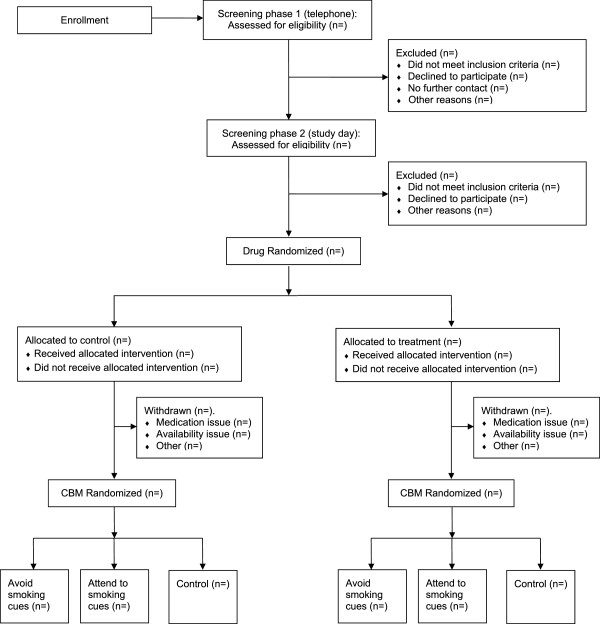
**CONSORT flow diagram.** CBM, cognitive bias modification.

Current smokers (n =72; 50% male) will be recruited from the staff and students at the University of Bristol (United Kingdom) and the general population. A CONSORT diagram of participant recruitment is shown in Figure 1. Participants will be recruited through existing email lists and poster and flyer advertisements, and by word of mouth. Participants who are interested in taking part will be sent the study information sheets and a telephone screening will be arranged to assess basic eligibility. Those who meet the study inclusion criteria will be booked in for two sessions approximately one week apart. Participants will receive £70 compensation for their time for participation. Full written informed consent will be obtained from each participant at the start of the study session. Participants will be given a copy of the information and consent form to keep.

### Study setting

All study sessions will be run at the Clinical Research and Imaging Centre at the University of Bristol, United Kingdom.

### Inclusion criteria

For inclusion, participants should be aged between 18 and 40 years, smoke at least 10 manufactured or 15 roll-up cigarettes per day, and smoke within one hour of waking in the morning. They should report availability to attend all of the study sessions and be able to adhere to all study requirements (including adhering to the seven-day drug administration regimen). Participants should have English as a first language, or have an equivalent level of fluency, and be able to give informed consent.

### Exclusion criteria

Participants will be excluded from this study if they have current or past alcohol or drug misuse and/or dependence, psychiatric illness, or clinically significant abnormality (including cardiovascular risk factors). Participants should not have ongoing use of any medication (at least eight weeks clear of any prescribed medication). Female participants should not be pregnant, breastfeeding, or at risk of becoming pregnant (not using adequate contraception). All participants must be registered with a GP, should not consume more than eight caffeinated beverages per day or more than 35 alcoholic units per week if female, or 50 alcoholic units per week if male (1 unit = a half pint (284 ml) of ordinary strength (3.5 to 4% alcohol-by-volume (ABV)) beer, a small glass (125 ml) of 8% ABV of wine, or a single measure (25 ml) of a 40% ABV spirit). They should not be actively trying to give up smoking and not be planning to quit during the study period. They should not have an uncorrected visual (including colour blindness) or auditory impairment or any known hypersensitivity to varenicline. Finally, they should not have any condition that would make magnetic resonance imaging (MRI) scanning unsafe or intolerable (such as metallic implants).

### Ethical considerations and informed consent

Ethics approval was obtained from the National Research Ethics Service (London Brent Committee; REC reference: 11/LO/1726). The study will be conducted according to the Declaration of Helsinki (sixth revision) and Good Clinical Practice guidelines. The investigator or co-investigator will explain the nature, purpose, and risks of the study to the participant. The participant will receive the information sheet in advance of the study sessions. There will be no time restriction on how long participants take to respond, with the exception that participants who respond after all study places have been filled will not be offered a place on the study. Therefore, participants will be given sufficient time to read the information and consider any implications, and to raise any questions with the investigators prior to making a decision to participate. Participants will be informed that they are free to withdraw at any time.

### Sample size determination

As our primary objective is to investigate the effects of CBM training on neural response to smoking-related cues, the sample size determination was based on this outcome. However, the effects of cognitive bias on neural responses to smoking cues have not been evaluated in previous research. To estimate the required sample size we used data from our previous study [19] that indicated a likely increase in the cognitive bias index of 30 ms in the attend group, and a decrease of 30 ms in the avoid group. We assume that the change in the control condition will be intermediate (0 ms). We will therefore achieve greater than 80% power to detect a linear effect across the three CBM groups on change in cognitive bias index with a total sample size of n = 30, at an alpha level of 0.05. Using data from a study that compared craving in response to smoking cues in groups who had received treatment of varenicline (mean = 11.2, standard deviation = 4.7) versus placebo (mean = 14.6, standard deviation = 4.7), we estimate a total sample size of 64 is required to achieve 80% power at an alpha level of 0.05. As we expect some attrition, we will use a more conservative estimate of n = 72.

### Randomization

Participants will be randomly assigned to drug and CBM groups, but equal numbers of participants per group will be maintained. The drugs will be prepared by University Hospitals Bristol Pharmacy who will produce two batches of 36 bottles (one for males and one for females) that comprise 18 bottles of varenicline and 18 bottles of placebo. Each bottle will be given a numeric identifier that enables study staff involved in data collection to be fully blinded to drug condition.

In advance of the study, an experimental collaborator (who will have no direct contact with the study participants) will prepare a numeric code using random number assignment software to further randomize participants to CBM groups. Randomization will be stratified so that equal numbers of male and female participants will be allocated to each experimental cell.

### Drug administration

Following initial consent and screening on day zero, varenicline (or matched placebo) will be prescribed for one week, to be taken as 0.5 mg once daily for days one to three, as 0.5 mg twice daily for days four to six, and as 0.5 mg once daily for day seven, consistent with the standard dosing regimen for smoking cessation. Participants will attend their second session on day seven (their last drug intake day) and will be advised to take the drug in the morning prior to their study session.

Study medication will be administered to the participant by a designated medical doctor following screening on day zero. This method of prescription is comparable with the administration of varenicline in general practice. Participants will be required to complete a daily diary detailing the time at which drugs are taken and any side effects. They will be asked to bring the diary and drug bottle to their second session.

### Outcome measures

Primary outcome measures are changes in cognitive bias as measured by the visual dot-probe task, and neural responses to smoking-related cues. Secondary outcome measures will be cognitive bias as measured by a transfer task (modified Stroop test) and subjective mood and cigarette craving as measured by the Questionnaire of Smoking Urges (QSU) and the Minnesota Nicotine Withdrawal Scale (MNWS). Measures and materials.

#### Materials

Stimuli for the CBM and fMRI cue exposure task comprise 32 full-colour smoking-related pictures and 32 neutral pictures. Smoking-related cues consist of full-colour pictures of people smoking. Control cues consist of full-colour pictures of people engaged in everyday activities (such as talking on the telephone or writing). Equal numbers of females and males are represented in each category. The set of cues pictures is the same as those used in previous imaging studies [26]. For the cognitive bias modification task, an additional four picture pairs, unrelated to smoking, are used in practice and buffer trials.

#### Cognitive bias modification

For the CBM, participants will be randomized to complete a version of the visual probe task designed to induce a biased cognitive response away from (avoid: n = 24), or towards (attend: n = 24) smoking-related cues, or a control condition (control: n = 24) designed not to modify cognitive bias. Each task version comprises a total of 768 trials. On each trial, following a 500 ms duration fixation cross, a picture pair is presented on a computer screen for 500 ms. When this picture pair disappears, a probe is presented in either of the two screen locations previously occupied by a picture. This probe is either a small circle or square. Participants are required to discriminate between the identity of each probe by pressing the arrow keys on the keyboard as appropriate, and the response latency is measured. The majority (n = 512) of trials are training trials, presented in four blocks, and the remainder (n = 256) of trials are test trials. Half of the test trials (n = 128) are presented prior to the training trials, and half (n = 128) after the training trials, in order to assess the effect of the training trials on cognitive bias. In the test trials, the probe appears with equal frequency in the vicinity of both the smoking-related or neutral pictures. In the training trials, the probe appears in the vicinity of the neutral picture on 75% of trials in the avoid condition, or appears in the vicinity of the smoking-related picture on 75% of trials in the attend condition, or appears with equal frequency in the vicinity of neutral and smoking-related pictures in the control condition. The inter-trial interval is jittered between 750 ms and 1,250 ms in order to reduce the monotony of the task. The sequence of events will be controlled using EPrime version 2 software (Psychology Software Tools Inc., Pittsburgh, Pennsylvania, United States), and the total task time is approximately 50 minutes.

#### Cognitive bias test (modified Stroop)

A pictorial version of the modified Stroop task will be used in favor of the more traditional lexical version, due to recent evidence of greater internal reliability of pictorial versions of the task [27]. The task begins with 16 practice trials followed by two experimental blocks, each comprising eight buffer and 96 experimental trials (208 trials in total). For each trial a picture is presented (smoking-related or neutral) centrally on screen. The picture is surrounded by a colored border and the participant is required to identify the colour of the border (red, blue, yellow, or green) using colour-marked keys on the keyboard (d, f, j, and k).

#### Questionnaires

The questionnaire measures will include the Eysenck Personality Questionnaire - Revised (EPQ-R) [28], the Questionnaire of Smoking Urges - Brief (QSU-Brief) [29], the Minnesota Nicotine Withdrawal Scale (MNWS) [30], and visual analogue scales (VAS) of mood and cigarette craving. These data will be used to examine group differences at baseline and to assess any changes in mood or craving across the course of the study.

#### functional Magnetic Resonance Imaging(fMRI) acquisition

**Figure 2:**
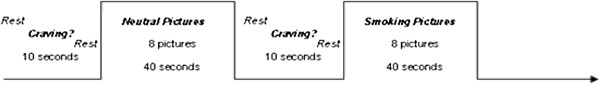
**Timeline of cue-exposure task conducted during the functional Magnetic Resonance Imaging.**

An anatomical and an fMRI scan will be performed on the test day session (day seven). During the fMRI cue exposure procedure, smoking-related and control cues will be presented in a boxcar design with four blocks per category (Figure 2). Participants are required to make a button press on each stimulus presentation to identify they have seen the image (this does not terminate viewing time). Each block will be 40 seconds in length, during which time eight cues will be presented. Before and after each block, a crosshair will be presented for five seconds. Participants will then be asked to rate their craving level on an eight-point scale (‘none at all’ to ‘extreme’). The scale will be presented for 10 seconds followed by presentation of a crosshair for another 10 seconds. Thus, the total between-block interval is 25 seconds. The sequence of events will be controlled using EPrime version 2 software (Psychology Software Tools Inc., Pittsburgh, Pennsylvania, United States), and the total task time is approximately 10 minutes. The total MRI protocol will last approximately 15 minutes.

### Data collection procedures

Day zero will be for screening and baseline assessments. After informed consent has been obtained, the screening procedure will be conducted. Expired breath alcohol and carbon monoxide readings will be taken and height, weight, blood pressure, and heart rate measured. A urine screen will be performed to test for recent drug use (all) and pregnancy (females). A medical doctor will perform a general physical and psychiatric health assessment and prescribe the study medication if appropriate. Post-screening, participants will complete a baseline assessment of cognitive bias (modified Stroop) and questionnaires assessing personality, cigarette craving, and mood. They will also complete a practice version of the task that will be used during the fMRI scan at the second study visit.

Participants will be sent away with the study medication, medication packaging information, and a drug diary (which they will be required to complete and return at the next visit). The second session (test day) will be scheduled for approximately one week later. This session will fall on day seven of their drug regimen.

On the test day, participants will complete the modified Stroop task followed by a short visual dot-probe task that measures baseline cognitive bias. They will then be randomized to one of three conditions: 1) an experimental condition designed to induce cognitive bias towards smoking-related cues (attend), 2) an experimental condition designed to induce cognitive bias away from smoking-related cues (avoid), or 3) a control condition designed to induce no change in cognitive bias (control). The test version of the dot-probe task will be run again immediately post-CBM in order to assess changes in cognitive bias.

Following this, participants will be taken to the scanning suite. All participants will complete a four-minute anatomical scan followed by the cue-exposure test during a 15-minute fMRI scan. After scanning, participants will complete the modified Stroop task and questionnaire state measures (QSU and visual analogue scales). At the end of the test session, participants will be offered smoking cessation literature, debriefed, and reimbursed (£70).

### Statistical plan

#### functional Magnetic Resonance Imaging analysis

Analyses will focus on evaluating neural activation during viewing of smoking-related and control cues in the three experimental groups. Pre-processing will be conducted using statistical parametric mapping software (Statistical Parametric Mapping 5; Wellcome Department of Imaging Neuroscience, London, United Kingdom) to remove noise and artefacts. The first four volumes of each run will be discarded to allow for T1 stabilization. All functional images will be corrected for acquisition timing and head motion using rigid-body rotation and translation. Each participant’s data will then be warped into a standard stereotaxic space (Montreal Neurological Institute) with an isotropic 2 mm voxel size and smoothed with an 8 mm full width at half measure (FWHM) Gaussian filter.

Each participant’s fMRI data will then be entered into a first-level voxel-by-voxel analysis using the general linear model. Each cue block (smoke or control) will be modelled as a boxcar function convolved with a canonical hemodynamic response function that begins at the onset of the first cue in the block and ends at the end of the block (60-second duration). A high-pass filter will be applied to remove slow signal drift. A smoking-related cue greater than control cue contrast image will then be created and inputted into a random effects analysis. A two (varenicline and placebo) × three (attend, avoid, and control) mixed-model analysis of variance (ANOVA)will be used to examine smoking cue reactivity (smoking greater than control) between each group. Voxel-wise analysis will be conducted within an Region of Interest (ROI) mask. The mask will be created from anatomical regions identified in a meta-analysis of cue reactivity [31]: nucleus accumbens, caudate, putamen, temporal gyrus, anterior cingulate gyrus, amygdala, insula, posterior cingulate cortex, inferior frontal gyrus, and angular gyrus. Each bilateral region will be selected using the WFU PickAtlas tool [32] and combined to create the mask. Resulting activations will be considered significant at an alpha level of 0.001 (uncorrected) with a minimum cluster extent threshold of 20 contiguous voxels. *Post hoc* analyses of parameter estimates will be used to evaluate the nature of significant effects. Smoking cue greater than control cue contrast images for each participant will also be input into random effect regression analyses examining relations between post-training cognitive bias scores and neural cue reactivity.

#### Cognitive bias analyses

Cognitive bias scores will be calculated from reaction time (RT) data from the visual probe task, by subtracting RTs to probes that replace smoking-related pictures from RTs to probes that replace neutral pictures, so that positive scores represent a bias towards smoking cues and negative scores represent a bias towards neutral cues. These bias scores will be used to examine cognitive training effects in a two (pre- and post-CBM) × two (varenicline and placebo) × three (attend, avoid, and control) mixed-model ANOVA.

A two (smoking cue and neutral cue) × two (pre- and post-CBM) × two (varenicline and placebo) × three (attend, avoid, and control) mixed-model ANOVA will also be conducted on the modified Stroop task RT and error data. Participants will be excluded if their mean RT or error score is three standard deviations (or more) below or above the sample mean.

#### Questionnaire analyses

To assess group differences at baseline two (varenicline and placebo) × three (attend, avoid, and control) between-subject ANOVAs will be conducted on EPQ-R, QSU, MNWS and VAS data. Mood (VAS) and craving (QSU and VAS) data will be analyzed in two time-phase analyses using ANOVA. We will first assess general (i.e., not cue-related)cravingacross drug treatment using baseline data from sessions one and two in a two (pre- and post-drug treatment) × two (varenicline and placebo) mixed-model ANOVA. We will also examine craving and mood change across CBM in a two (pre- and post-CBM) × two (varenicline and placebo) × three (attend, avoid, and control) mixed ANOVA, in which time is a within-subjects factor and drug is a between-subjects factor.

## Discussion

This study will investigate the neural effects of smoking-related CBM in daily smokers. We will also examine whether one-week’s treatment of the licensed smoking cessation pharmacotherapy varenicline alters neural response to smoking-related cues. Within this data set, we will be able to explore whether there is any pattern suggesting an interactive effect of CBM and varenicline. These are novel questions in this field and will inform future research with a better understanding of the mechanisms that may underlie CBM. As CBM is a treatment which can be delivered remotely via computers, tablets, or smartphones it could therefore provide a cost-effective intervention that could be used, in conjunction with more traditional pharmacotherapies and behavioral support, to help promote long-term smoking cessation and reduce relapse rates.

This study will recruit daily smokers who are not currently trying to quit smoking. Therefore this is an analogue sample and participants have not been recruited on the basis of seeking cessation support. The primary reason for this is due to the proposed mechanism by which varenicline impacts on cue reactivity. Although this mechanism has not been unequivocally established, it is suggested that smoking cues lose incentive salience during varenicline treatment due to its attenuation of smoking reinforcement. That is, individuals experience reduced reward from smoking and this is associated with drug-related cues. For this process to occur however, individuals need to smoke to experience the attenuated smoking reinforcement. For the purposes of this study, it was not considered ethical to enroll smokers motivated to quit as their participation may maintain smoking and delay a quit attempt. Recruitment of non-quitting smokers also avoids interference of other smoking-related treatments such as nicotine replacement therapy or behavioral support. This study will provide a basis to examine treatment effects of varenicline and CBM on cognitive bias and cue-related responses that can inform future research in quitting populations.

## Trial status

As of March 2014, 55 participants have been enrolled onto the study. The first participant was enrolled in November 2012 and completion of data collection is projected for June 2014.

## Abbreviations

ANOVA: Analysis of variance
CBM: Cognitive bias modification
EPQ-R: Eysenck personality questionnaire-revised
FWHM: full width at half maximum
fMRI: functional Magnetic Resonance Imaging
MNWS: Minnesota nicotine withdrawal scale
NRT: Nicotine replacement therapy
QSU: Questionnaire of smoking urges
RT: Reaction time
ROI: Region of interest
SPM: Statistical parametric mapping
VAS: Visual analogue scales.
